# Autophagy and its link to type II diabetes mellitus

**DOI:** 10.1051/bmdcn/2017070201

**Published:** 2017-06-14

**Authors:** Jai-Sing Yang, Chi-Cheng Lu, Sheng-Chu Kuo, Yuan-Man Hsu, Shih-Chang Tsai, Shih-Yin Chen, Yng-Tay Chen, Ying-Ju Lin, Yu-Chuen Huang, Chao-Jung Chen, Wei-De Lin, Wen-Lin Liao, Wei-Yong Lin, Yu-Huei Liu, Jinn-Chyuan Sheu, Fuu-Jen Tsai

**Affiliations:** 1 Department of Medical Research, China Medical University Hospital, China Medical University Taichung 404 Taiwan; 2 School of Pharmacy, China Medical University Taichung 404 Taiwan; 3 Department of Biological Science and Technology, China Medical University Taichung 404 Taiwan; 4 Genetics Center, Department of Medical Research, China Medical University Hospital Taichung 404 Taiwan; 5 School of Chinese Medicine, China Medical University Taichung 404 Taiwan; 6 Institute of Biomedical Sciences, National Sun Yat-sen University Kaohsiung 804 Taiwan; 7 Department of Medical Genetics, China Medical University Hospital, China Medical University Taichung 404 Taiwan

**Keywords:** Autophagy, Type 2 diabetes mellitus (T2DM), Pancreatic β-cells, Insulin resistance

## Abstract

Autophagy, a double-edged sword for cell survival, is the research object on 2016 Nobel Prize in Physiology or Medicine. Autophagy is a molecular mechanism for maintaining cellular physiology and promoting survival. Defects in autophagy lead to the etiology of many diseases, including diabetes mellitus (DM), cancer, neurodegeneration, infection disease and aging. DM is a metabolic and chronic disorder and has a higher prevalence in the world as well as in Taiwan. The character of diabetes mellitus is hyperglycemia resulting from defects in insulin secretion, insulin action, or both. Type 2 diabetes mellitus (T2DM) is characterized by insulin resistance and failure of producing insulin on pancreatic beta cells. In T2DM, autophagy is not only providing nutrients to maintain cellular energy during fasting, but also removes damaged organelles, lipids and miss-folded proteins. In addition, autophagy plays an important role in pancreatic beta cell dysfunction and insulin resistance. In this review, we summarize the roles of autophagy in T2DM.

## 1. Introduction

**Fig. 1:**
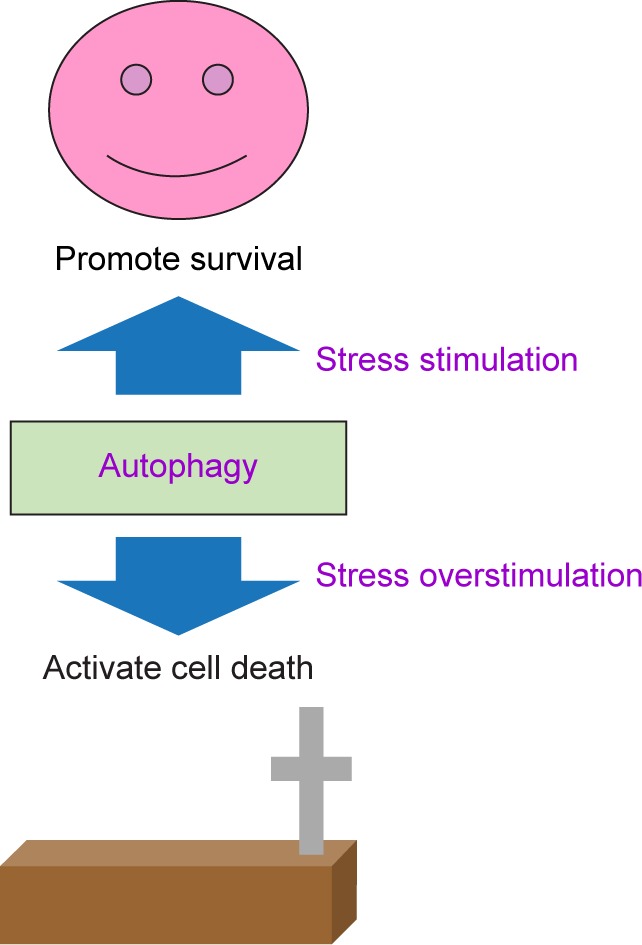
Autophagy serves as a double-edged sword. Autophagy promotes survival character when cells undergo stimuli and/or activates cell death when stimuli exceed a threshold.

Professor Yoshinori Ohsumi, the 2016 laureate in Physiology or Medicine, discovered the mechanisms for autophagy [1–4]. This pathway plays a crucial role in physiological cellular homeostasis and human diseases [5]. Autophagy has been known to serve as a double-edged sword for promoting survival character and/ or activating cell death (Fig. 1) [6–11]. In addition, autophagy, a catabolic process, degrades cellular components and damaged organelles [12, 13]. Recently, autophagic machinery is involved in the pathophysiology of type 2 diabetes mellitus (T2DM) disease, and it regulates normal function of pancreatic beta cells. On the other hand, enhanced autophagy acts as an important protective mechanism against to oxidative stress on insulin-target tissues such as liver, adipose tissue and skeletal muscle [14–19]. In this review, we outline the relationship among autophagy, pancreatic beta cells and T2DM. Furthermore, we highlight recent findings on the novel agents to specifically target autophagy in T2DM.

## 2. Programmed cell death (PCD)

PCD is an important physiological process during organ development, tissue homeostasis. This process is a protective mechanism against cellular stress, drug, external environment and tumor suppressive mechanism. It is generally divided into three distinct types including: (1) apoptosis; (2) autophagic cell death; and (3) necroptosis. Each type of cell death exhibits the specific morphological, molecular and biochemical characteristics [20]. We summary the characteristics of the three types as listed in Table 1.

**Table 1:** The characteristic features of programmed cell death [20].

Programmed cell death (PCD)	Apoptosis (type I PCD)	Autophagic cell death (type II PCD)	Necroptosis (type III PCD)
Feature	Chromatin condensation	Autophagic vesicles	Random DNA degradation
	DNA laddering	Blebbing	Swollen organelles
	Blebbing (nuclear, cytoplasmic)	Degradation of golgi	Cytoplasmic membrane rupture
	Apoptotic bodies		Potent inflammatory response
Key regulators	Caspases	Beclin-1	RIPK1
	Bcl-2 family members	LC III	TRAF2
	Cytochrome c	Atg family proteins	PARP
	AIF		
	Death-receptor proteins	ULK 1	Calpains
	Calpains	mTOR	
Relative pathways	Death-receptor Pathway (extrinsic pathway)	AMPK pathway	Glycosylphospha-tidylinositol anchor biosynthesis
	Mitochondrial pathway (Intrinsic pathway)	Akt/mTOR pathway	Type 1 interferon family
	ER stress pathway	MAPK/ERK pathway	
	Caspase-dependent pathway	p53/stress pathway	Toll-like receptor signaling network
	Caspase-independent pathway	ER stress pathway	

Apoptosis (Type I PCD) is characterized by chromatin condensation, DNA fragmentation and laddering, blebbing of nuclear or cytoplasmic and apoptotic bodies [21]. Apoptotic pathways include death-receptor pathway (extrinsic pathway), mitochondrial pathway (intrinsic pathway), endoplasmic reticulum (ER) stress, caspase-dependent pathway and caspase-independent pathway [22–27]. In the death-receptor pathway (extrinsic pathway), cell death is mediated by the interaction between death receptor proteins (such as Fas/CD95, DR4 and DR5) and the ligand (such as FasL and TRAIL), resulting in the staffing of an adaptor protein (FADD) and activation of caspase-8 and caspaswe-3/7 [22–32]. Mitochondria plays an essential role in the intrinsic pathway, which is inactivated by a drug or stress and then disrupts the mitochondrial membrane potential, causing production of reactive oxygen species (ROS) and release of cytochrome c, Apaf-1, procaspase-9, AIF and Endo G signaling. The cytochrome c, Apaf-1 and procaspase-9 form an apoptosome complex to activate caspase-9 and caspase-3/-7. In addition, pro-apoptotic Bcl-2 family proteins (such as Bax, Bak, Bim, Bid, *etc.)* and anti-apoptotic proteins (such as Bcl-2, Bcl-xL, Mcl-1, *etc.)* regulate the process of mitochondrial pathway [33–41]. ER stress is induced by accumulation of unfolded/misfolded protein aggregating in ER or by excessive protein traffic. Increasing the proteins level of GADD 153, GRP 78, GRP 94 and ATF6, the hallmarks of ER stress, induce a rise in intracellular Ca^2+^ level, mitochondrial membrane depolarization and activation of calpain and caspase-12 in murine systems and/or caspase-4 in human cells [29, 36, 42–45].

**Fig. 2:**
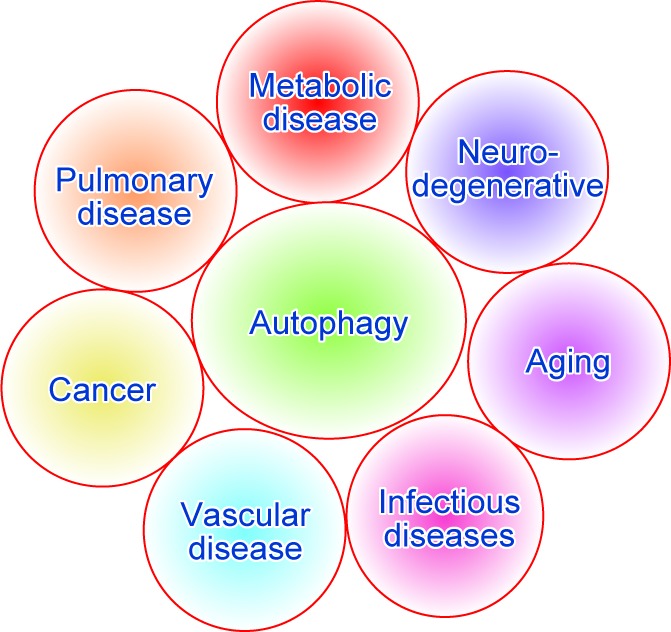
Autophagy participates in the pathogenesis of human diseases. These human disorders include DM, neurodegenerative diseases, aging, pathogen infection diseases, vascular disease, pulmonary disease and cancer.

Autophagic cell death (type II PCD) is a process by eliminating intracellular components through the lysosomal degradation in eukaryotic cells. Autophagy was first discovered during the late 1950s and early 1960s [46–48]. In the 1990s, the essential genes of the autophagy pathway were identified and characterized by the genetic screen studies in baker’s yeast [49, 50]. Autophagy has been demonstrated to be involved in many biological processes, including maintenance of organelle integrity, protein quality control, regulation of the stress response and immune response [51–62]. Recently, autophagy has been shown to be modulated and to participate in the pathogenesis of human diseases, such as DM, neurodegenerative diseases, aging, pathogen infection diseases, vascular disease, pulmonary disease and cancer (Fig. 2) [13, 63–68]. Dr. Yoshinori Ohsumi discovered autophagy-related genes (ATGs) using a genetic screening approach in *Saccharomyces cerevisiae* and awarded the 2016 Nobel Prize in Physiology or Medicine for his remarkable contribution to autophagy research [1, 4, 69–71].

Autophagy is characterized by an increase of double-membrane vesicles (also known as autophagosomes or autophagic vesicles) and degradation of golgi. Autophagy promotes cell survival in response to stress; however, once autophagy is overstimulated, cells can progress to autophagic cell death (Fig. 1). Here, we propose clearer definitions of the roles on autophagy: (A) the first role of autophagy functions as cell survival or cell protection [72–75]. (B) the second role of autophagy mediates programmed cell death (autophagic PCD). Upon stress, early-onset autophagy triggers cell protection and then late-onset autophagy induces cell death [76–80]. The detailed molecular mechanisms of autophagy will be described later.

Necroptosis (type III PCD), an irreversible cell death [81, 82], is characterized by a gain in cell volume, swollen organelles, DNA degradation, cytoplasmic plasma membrane rupture, subsequent loss of intracellular contents and potent inflammatory response. Relative necroptosis pathways include glycosylphosphatidylinositol anchor biosynthesis pathway, type 1 interferon family pathway and toll-like receptor signaling pathway (Table 1) [20]. The protein kinase RIP1 and RIP3 are central molecules in necroptosis. The RIPK1, TRAF2, PARP, calpains and RIPK3 proteins are identified and associated with programmed necrosis [83–89].

## 3. Assays for monitoring autophagy and pharmacological regulated agents

The features of autophagy are the massive accumulation of autophagic vacuoles (autophagosomes) in the cytoplasm of cells. Hereby, we present a series of methods to monitoring autophagy in Table 2. (1) Transmission electron microscopy (TEM) is used to observe autophagosome number, volume, and content analysis; (2) The lysosomal enzymes activity, assessment of the number, size, and location of lysosomes are examined by the uptake of fluorescent dyes (monodansylcadaverine (MDC), acridine orange (AO), neutral Red, LysoSensor Blue, Lyso-Tracker Red); (3) Autophagy-related proteins such as ATGs and LC3 are detected by western blotting or fluorescent protein tagging; (4) Autophagy-related gene expression levels are measured by western blotting or real-time PCR. Table 3 is a list of the pharmacological agents for assessing autophagy effects such as inhibition of lysosomal enzyme activities, fusion of organelles, or inter-compartmental transfer of molecules. (1) The 3-methyladenine (3-MA) is a PtdIns3K inhibitor and blocks an early stage of autophagy. (2) Bafilomycin A1 is a V-ATPase inhibitor and blocks fusion of autophagosomes with the vacuole. (3) Chloroquine is a lysosomotropic compound that elevates and neutralizes the lysosomal and vacuolar pH. (4) Leupeptin blocks lysosomal protein degradation. (5) Pepstatin A inhibits lysosomal protein degradation. (6) Resveratrol induces autophagy through activation of AMPK and (7) Tunicamycin is a glycosylation inhibitor that induces autophagy [55, 90, 91].

**Table 2:** Assays for monitoring autophagy.

Description	Methods	Reference
Monitor autophagosome number, volume, and content/cargo	Transmission electron microscopy (TEM)	[59, 146, 147]
	Western blotting	[59, 146, 147]
Atg8/LC3 detection and quantification	GFP-Atg8/LC3 fluorescence microscopy	[59, 146, 147]
	Immunohistochemistry	[59, 146, 147]
	Western blotting	[55, 56, 59, 60, 62, 146, 147]
Additional autophagy-related protein markers	Real-time PCR	[55, 56, 59, 60, 62, 146, 147]
	Immunohistochemistry	[55, 56, 59, 60, 62, 146, 147]
Transcriptional regulation	Real-time PCR	[55, 56, 59, 60, 62, 146, 147]
	Monodansylcadaverine (MDC)	[59, 146, 147]
	Acridine orange (AO)	[59, 146, 147]
Acidotropic dyes for identify acidified vesicular compartments	Neutral Red	[59, 146, 147]
	LysoSensor Blue	[59, 146, 147]
	Lyso-Tracker Red	[59, 146, 147]

**Table 3:** Pharmacological regulation of autophagy.

Method	Comments	Reference
3-Methyladenine (3-MA)	The PtdIns3 K inhibitor and blocks an early stage of autophagy	[60, 62, 90, 91]
Bafilomycin A1	The V-ATPase inhibitor and blocks fusion of autophagosomes with the vacuole	[58, 62, 90, 91, 148]
Chloroquine	Lysosomotropic compounds that elevate and neutralize the lysosomal and vacuolar pH	[58, 90, 91]
Leupeptin	Block lysosomal protein degradation	[90, 91]
Pepstatin A	Block lysosomal degradation	[90, 91]
Tunicamycin	The glycosylation inhibitor that induces autophagy	[90, 91]
Resveratrol	Induction of autophagy via activation of AMPK	[55, 90, 91]

## 4. The molecular mechanisms of autophagy

**Fig. 3:**
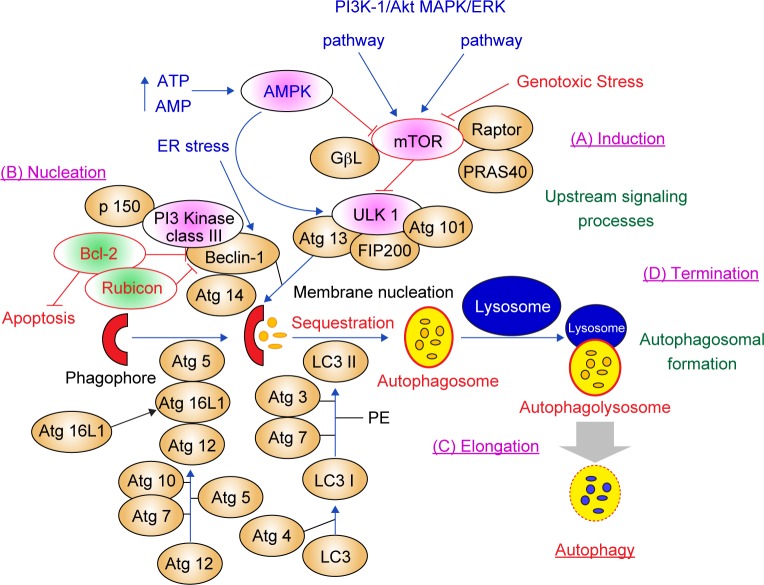
There are four stages in the autophagic process: (1) induction, (2) nucleation, (3) elongation and (4) termination.

There are four stages in the autophagic process: (1) induction, (2) vesicle nucleation, (3) autophagosome membrane elongation and (4) termination/ fusion and degradation l (Fig. 3) [92, 93]. In the normal status such as adequate nutrition, the mTORC1 complex (mTOR/GpL/Raptor/PRAS40) interacts with the ULK1 complex (ULK1/2-Atg13-FIP200-Atg101) to inhibit autophagy. When the mTORC1 complex senses genotoxic stress from hypoxia, starvation and low energy levels, mTORC1 dissociates from the ULK1 complex and initiates autophagy. Recent evidence suggests that mTORC1 complex is also regulated by PI3K-1/Akt, MAPK/ ERK and AMPK signaling pathway. Activated AMPK phosphorylates Raptor and inhibits mTOR, which leads to activation of autophagy [94–98].

Beclin-1 complex (PI3Kinase class III, p150, Beclin-1 and Atg14) is essential for vesicle nucleation and stimulates the fusion of autophagosomes with lysosomes [94–98]. During the stage of vesicle nucleation, Beclin-1 interacts with Atg14L, Bcl2, Rubicon, p150 and PI3Kinase class III proteins. Several regulators such as Bcl-2 protein (anti-apoptotic protein) and Rubicon bind Beclin-1 and inhibit the vesicle nucleation stage of autophagy.

Autophagosome membrane engagement is executed by the Atg12 and LC3 ubiquitin-like conjugation systems. (1) Atg12 ubiquitin-like conjugation system: ubiquitin-like Atg12 is conjugated to Atg5, Atg7 and Atg10. Atg10 serves as the E2 enzyme. The Atg5-Atg12/Atg16L complex is regulated by the Beclin-1 complex and localizes to the convex surface of the isolation membrane. (2) LC3 ubiquitin-like conjugation system: LC3 is cleaved by the Atg4 cysteine protease, sequentially processed by Atg7 and Atg3 and then conjugated to the membrane lipid phosphatidylethanolamine (the conjugated form is termed LC3-II). The Atg5-Atg12/Atg16L1 complex is necessary to promote the transformation of LC3-I to LC3-II [94–98].

At the terminal stage of autophagy, the autophagosome fuses with lysosomes to form autophagolysosomes. Autophagy allows the orderly degradation and recycling of cellular components [99]. The purpose of autophagy is to ensure quality control of organelles and proteins, as well as protection of intracellular homeostasis in stress and nutrient efficiency [94–99].

## 5. Type 2 diabetes mellitus (T2DM)

Diabetes mellitus (DM), commonly referred to as diabetes, is a metabolic and chronic disease in the world [100, 101]. DM patients have high blood sugar levels over a prolonged period. The character in DM is a relative or absolute lack of insulin, resulting in hyperglycemia [102]. Symptoms of hyperglycemia are frequent urination, increased thirst, and increased hunger. Acute complications of DM can include nonketotic hyperosmolar coma, diabetic ketoacidosis and death. Serious complications of DM include cardiovascular disease, stroke, chronic kidney failure, nephropathy, foot ulcers, neuropathy and damage to the eyes [103–106]. In 2014, approximately 422 million people were diagnosed with DM according to World Health Organization (WHO) report [107, 108]. In Taiwan, DM is ranked as the fifth leading cause of death in 2015 on the basis of statistics by the Ministry of Health and Welfare, R.O.C. (Taiwan) [101, 109].

There are three main types of diabetes mellitus: (1) Type 1 diabetes (T1D): also called insulin-dependent, juvenile or childhood-onset diabetes. T1D is characterized by deficient insulin production in the body. The pathology in T1D is described as an autoimmune disease because the pancreatic beta cells (insulin-producing tissue) are destructed in the islets of Langerhans [110]. T1D is diagnosed most in children and young adults. People with T1D require daily administration of insulin to regulate the amount of glucose in their blood [111]. Environmental factors and genetic influence play an important role in T1D [112, 113]. (2) Type 2 diabetes (T2D): formerly called non-insulin-dependent (NIDDM) or adult onset diabetes. T2D is the most common type of diabetes with prevalence in Taiwan. T2D begins with insulin resistance in which cells fail to respond to and uptake of insulin in the body [114–116]. Insulin resistance can be enhanced by weight reduction and exercise [117]. (3) Gestational diabetes: pregnant women without a previous history of diabetes develop high blood sugar levels [118, 119].

**Fig. 4:**
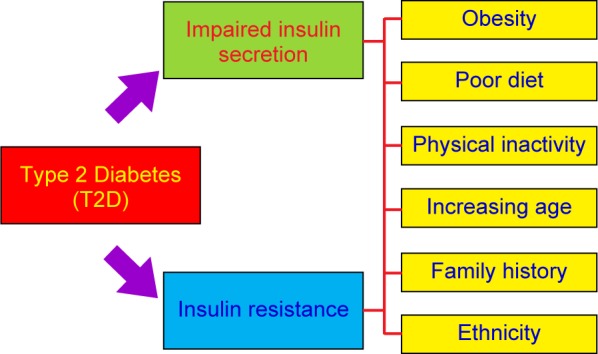
Etiology of type 2 DM. Two major physiological defects associated with T2D are reduced insulin sensitivity, insulin resistance and combined with impaired insulin secretion. Obesity, poor diet, physical inactivity, increasing age, family history and ethnicity lead to a higher risk of T2D.

**Fig. 5:**
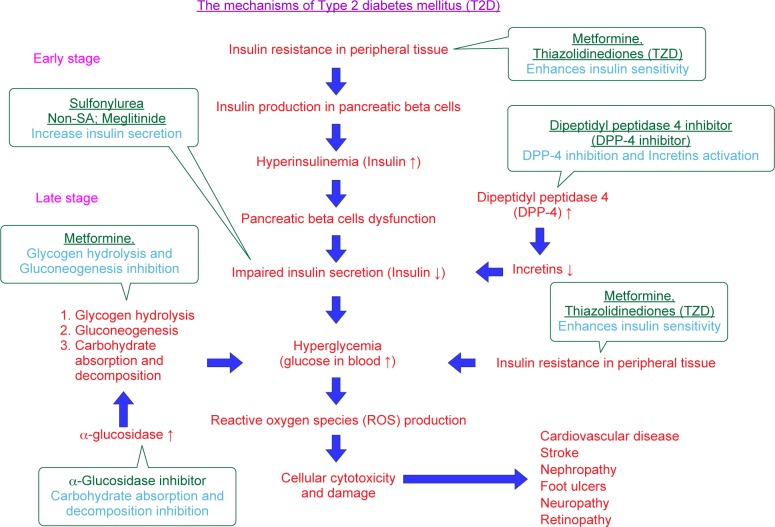
Mechanisms in the development and pharmacological treatments of T2D. Details are described in the text.

The physiological defects in T2D that is reduced insulin sensitivity, insulin resistance and combined with impaired insulin secretion (Fig. 4). T2D occurs as a result of obesity, poor diet, physical inactivity, increasing age, family history and ethnicity. The defective or mutant insulin receptor may be caused no response to insulin in body tissues. Controversially, patients with T2D in the early stage often have a normal or high bone mineral density (BMD), associated with obesity and hyperinsulinemia, as well as altered level of insulin. When cells are insensitive to insulin (or insulin resistance), the pancreatic beta cells produce more and more insulin, which leads to the higher insulin concentration in blood (hyperinsulinemia). The pancreatic beta cells desperately secrete insulin and then gradually decline. T2D at late stage is characterized by insufficient secretion of insulin from the pancreatic beta cells, coupled with impaired insulin action in target tissues such as muscle, liver and fat. Hyperglycemia results when insulin secretion is unable to compensate for insulin resistance [120–124]. Mechanisms in the development and pharmacological treatments of T2D are summarized in Fig. 5 and Table 4.

**Table 4:** Pharmacological treatment for T2D.

Mechanisms	Type	Drugs	Reference
Increase insulin secretion from pancreatic β-cells.	Sulfonylureas (First generation)	Tolbutamide Chlorpropamide Acetohexamide Tolazamide	[149–158]
	Sulfonylureas (Second generation)	Glibenclamide (Euglucon ®) Glipizide (Glidiab®) Gliclazide (Diamicron ®) Glimepiride (Amaryl®)	[149–158]
	Meglitinide	Repaglinide (Novonorm®) Nateglinide (Starlix®)	[159–162]
Enhances insulin sensitivity in liver and peripheral tissues by activation of AMP activated protein kinase. Glycogen hydrolysis and Gluco- neogenesis inhibition.	Biguanide	Metformin	[163–167]
Absorption of glucose is delayed.	α-Glucosidase inhibitor	Acarbose	[168–170]
Enhances insulin sensitivity in peripheral tissues and liver by activation of peroxisome proliferator-activated receptor-gamma receptors.	Thiazolidinedione (TZD)	Rosiglitazone (Avandia®) Pioglitazone (Actos)	[171–174]
Amplifies incretin pathway activation by inhibition of enzymatic breakdown of endogenous GLP-1 and GIP.	DPP-4 inhibitor	Sitagliptin (Januvia) Saxagliptin (Onglyza) Linagliptin (Trajenta)	[150, 175–178]
Activates incretin pathway by utilizing DPP-4 resistant analogue to GLP-1.	GLP-1 receptor agonist	Exenatide (Byetta) Liraglutide (Victoza)	[179–185]
Activates insulin receptors to regulate metabolism of carbohydrate, fat and protein.	Insulin Bolus (prandial) insulins Basal insulins Premixed insulins	Aspart (NovoRapid) Glulisine (Apidra) Lispro (Humalog) Detemir (Levemir) Glargine (Lantus) NPH (Humulin-N, Novolin ge NPH) Biphasic insulin aspart (NovoMix 30) Insulin lispro/lispro protamine suspension (Humalog Mix25, Mix50) Premixed Regular-NPH (Humulin 30/70; Novolin ge 30/70, 40/60, 50/50)	[111, 186-191]

## 6. Autophagy and type 2 diabetes (T2D)

Autophagy has been known to regulate the function of pancreatic beta cells and insulin-target tissues (skeletal muscle, liver and adipose tissue). T2D progression through impaired pancreatic beta cells function and development of insulin resistance has been associated with autophagy [125–128]. Upon insulin resistance, pancreatic cells enhance their insulin secretion (hyperinsulinemia) to compensate for hyperglycemia on the early onset of T2D (Fig. 5). In contrast, the number of pancreatic cells is progressive diminution through apoptotic cell death on the late onset of T2D [125, 129–131].

Many studies suggest that enhanced autophagy acts as a protective mechanism against oxidative stress in pancreatic beta cells [128, 132]. *In vivo* studies demonstrated that Atg7-deficient mice showed a decrease in the number of pancreatic beta cells, impairment of glucose tolerance and reduction in insulin secretion [133]. The insulin resistant mice (beta-cell-specific Atg7 knockout mice) model has been shown that autophagy plays a crucial role in the development of diabetes and in preserving the structure and function of pancreatic beta cells. Accumulation of autophagosomes in the pancreatic beta cell has been demonstrated in *db/db* mouse model [134–136]. Fujitani *et al.* showed that reduced insulin secretion was associated with pancreatic beta cell degeneration and impaired glucose in autophagy-deficient mice [136–138]. However, constitutively activated autophagy has injurious effects on pancreatic beta cells and chronic activation of autophagy causes autophagic cell death [135, 139–143].

## 9. Conclusion

**Fig. 6:**
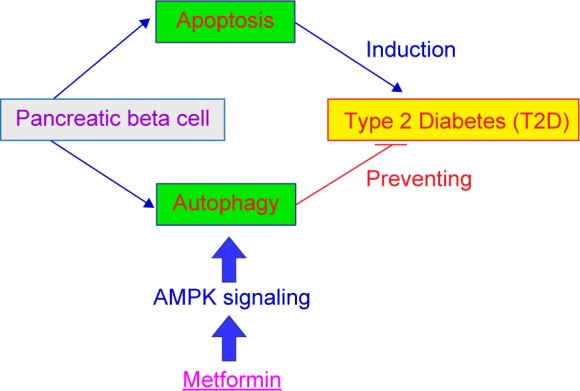
The role of autophagy and apoptosis in T2D.

The pancreatic beta cells control the releases of insulin and play an important role in the progression of T2D. Autophagy might function as a protective and pro-survival role on pancreatic beta cell death in T2D. Metformin has been widely used in the clinic therapy in T2D and has a protective effect on pancreatic beta cells from injury by activating autophagy through AMPK pathway [144, 145]. Therefore, it is urgent to understand the relationship of autophagy and T2D. We summarize the role of autophagy and apoptosis in T2D in Fig. 6. It is expected to develop new drugs and more effective agents targeted in autophagy for the therapy of T2D.
